# GW0742 as a Potential TRα and TRβ Antagonist Reduces the Viability and Metabolic Activity of an Adult Granulosa Tumour Cell Line and Simultaneously Upregulates TRβ Expression

**DOI:** 10.3390/cancers16234069

**Published:** 2024-12-05

**Authors:** Justyna Gogola-Mruk, Izabela Kumor, Gabriela Wojtaszek, Karolina Kulig, Anna Ptak

**Affiliations:** Laboratory of Physiology and Toxicology of Reproduction, Institute of Zoology and Biomedical Research, Jagiellonian University, Gronostajowa 9, 30-387 Krakow, Poland; izabela.kumor@student.uj.edu.pl (I.K.); gabriela.wojtaszek@student.uj.edu.pl (G.W.); karolina.m.kulig@student.uj.edu.pl (K.K.); anna.ptak@uj.edu.pl (A.P.)

**Keywords:** TRα and TRβ, GW0742, celecoxib, T_3_, rare type of ovarian cancer, ovarian granulosa cells, ATP production rate

## Abstract

Background/Objectives: Clinical studies have demonstrated a correlation between alterations in the expression level of TRα and TRβ receptors in ovarian cancer cells and overall survival. Celecoxib and GW0742, commonly known as a COX-2 inhibitor and a PPARβ/δ agonist, are novel thyroid hormone receptor antagonists that bind to TRβ or both TRα and TRβ. Methods: The study was conducted on a non-luteinized ovarian granulosa cell line (HGrC1) and two rare ovarian cancer cell lines (COV434 and KGN). The expression of TRα and TRβ at the gene and protein levels was examined by real-time PCR and Western blot, respectively. The impact of GW0742 and celecoxib on the cell viability of the HGrC1, COV434 and KGN lines was evaluated using the PrestoBlue™ Cell Viability Reagent. The metabolic activity of the cells was analysed using the Seahorse XFp Analyzer. Results: Initially, we observed that the gene and protein expression levels of TRα and TRβ were higher in COV434 and KGN cells than in HGrC1 cells. Subsequently, it was demonstrated that T_3_ enhances the viability of HGrC1, COV434 and KGN cells. Furthermore, autoregulatory feedback loops were not observed during TRα or TRβ signalling in ovarian cancer cells, in contrast to the findings in healthy granulosa cells. Finally, we demonstrated that GW0742 reduced the viability and metabolic activity of granulosa cell tumours (GCTs). Simultaneously, we observed that GW0742 upregulated the expression of TRβ in GCT. Conclusions: These findings suggest that GW0742 may be a novel adjuvant therapy for GCTs expressing TRα and TRβ.

## Simple Summary

Epidemiological data indicate an association between thyroid dysfunction and an increased risk of ovarian cancer. Furthermore, receptors for thyroid hormones (TRs) are shown to be expressed in ovarian cancer cells. In this study, we compared the expression of TRα and TRβ in human rare ovarian cancer cells (COV434 and KGN) with non-cancer ovarian granulosa cell line. We found higher expression of these receptors in cancer cells. Additionally, we demonstrated that triiodothyronine (T_3_) increases the viability and the expression of TRα and TRβ in rare of ovarian cancer cells which is probably due to the lack of autoregulation of the feedback loops during TR signalling. Our study suggests that the use of novel thyroid hormone receptor antagonists, such as GW0742, may play a role in reducing the viability and metabolism of ovarian cancer cells and may find future application in adjuvant therapy for adult granulosa cell tumours.

## 1. Introduction

Thyroid hormone receptors α and β (TRα and TRβ) belong to the superfamily of nuclear receptors that mediate thyroid hormone function in many tissues, where they regulate important physiological and developmental processes such as growth and metabolism [1]. Both TRα and TRβ are expressed in healthy ovarian tissues [2,3,4]. Interestingly, it has been shown that the expression of thyroid hormone receptors (TRs) changes during ovarian cancer tumorigenesis. Moreover, a higher expression level and localisation of TRα and TRβ in epithelial ovarian cancer cells are correlated with patient survival time [5,6]. Rasool et al. showed that women with ovarian cancer have higher levels of T_3_ and T_4_ than healthy women, indicating a link between hyperthyroidism and increased proliferation of ovarian cancer cells [7].

A significant problem in ovarian cancer (OC) research is the heterogeneity of the disease [8]. Approximately 90% of ovarian malignancies are epithelial tumours, whereas several rare tumours, including sex cord tumours and germ cell tumours, are categorised as non-epithelial tumours [9]. Among them, we can distinguish granulosa cell tumour (GCT) [10] and small-cell carcinoma of the ovary, hypercalcaemic type (SCCOHT) [11]. Rare types of OC are poorly characterised, without well-described standard and adjuvant treatments with published data, mainly as a case study [12]. Therefore, new perspectives for treating these histological types of OC are needed.

The use of nonsteroidal anti-inflammatory drugs that bind to TRα or TRβ and antagonise the effects of thyroid hormones is a novel concept in cancer therapy [13]. Targeting TR has already been reported as a therapy for colorectal cancer [14]. As both TRα and TRβ are expressed in different histological subtypes of ovarian cancer cells, it is crucial to evaluate their effects on rare OC and examine the potential use of TR inhibitors as targeted therapy in rare OC. Based on recent studies, two inhibitors can be selected as potential substances for use in OC therapy. Chemical analyses revealed that GW0742 (commonly known as a PPARβ/δ agonist) can bind and antagonise TRα and TRβ [15]. Another study by Zloh et al. showed that celecoxib (a COX-2 inhibitor) is an antagonist of TRβ [16]. Here, we hypothesise that TR antagonists can reverse the potential growth-promoting effects of T_3_ and reduce the viability and metabolic activity of rare OC.

Therefore, the primary aim of this study was to examine whether GW0742 (a TRα and TRβ antagonist) and celecoxib (a TRβ antagonist) reduce tumour cell viability and metabolic activity, simultaneously counteracting the proliferative effects of T_3_. First, we assessed the basal expression of TRα and TRβ in healthy ovarian granulosa cells (GCs) and rare OC (granulosa cell tumour and small-cell carcinoma of the ovary) and defined the effect of T_3_ on the viability of these cells. Next, we investigated the dose-dependent effect of GW0742 and celecoxib on the viability of rare OC and evaluated whether these compounds reverse the effects of T_3_ in the cells. Additionally, we analysed the metabolic activity by assessing the ATP production rate independent of the metabolic pathways. Lastly, we examined changes in the expression of TRα, TRβ, PPARβ/δ and COX-2 in the presence of GW0742 and celecoxib.

## 2. Materials and Methods

### 2.1. Chemicals

The test substances GW0742 (cat. nr: G3295) and celecoxib (cat. nr: SML3031) were purchased from Sigma-Aldrich (St. Louis, MO, USA), and 3,3′,5-triiodo-L-thyronine (cat. nr: S5726) was from Selleckchem Inc. (Houston, TX, USA) and subsequently dissolved in dimethyl sulfoxide (DMSO; cat. nr: 2650; Sigma-Aldrich). The final concentration of DMSO in the cell growth medium was <0.01% (*v*/*v*).

### 2.2. Cell Culture and Treatment

In vitro model systems were used to investigate the effects of GW0742, celecoxib and T_3_ on cell viability in rare types of human ovarian cancer: GCT, represented by the KGN cell line (Riken Cell Bank (RBRC-RCB1154), Ibaraki, Japan) and SCCOHT, represented by the COV434 cell line (European Collection of Authenticated Cell Cultures (ECACC) #07071909; Sigma-Aldrich) [17,18]. As an in vitro model of non-tumour ovarian cells, we used a non-luteinised ovarian granulosa cell line (HGrC1), which was a generous gift from Dr Ikara Iwase (Nagoya University, Japan). HGrC1 and COV434 cells were cultured in phenol red-free DMEM (Sigma-Aldrich) supplemented with 2 mM L-glutamine and 10% charcoal-stripped foetal bovine serum (FBS) (Biowest, Nuaille, France). KGN cells were cultured in DMEM/Ham’s F12 medium without phenol red (Thermo Fisher Scientific, Waltham, MA, USA) with 10% FBS. The cells were cultured in a consistently controlled environment in a closed incubator set at a temperature of 37 °C, with 5% CO_2_ and a relative humidity of 95%. HGrC1, COV434 and KGN cells were treated for 24 or 48 h with different concentrations of T_3_ (0.1, 1, 10 or 100 nM); GW0742 (1, 10, 100 ng/mL or 1, 10, 25 or 100 µg/mL) or celecoxib (0.1, 1, 5, 10 or 15 µg/mL). A cell viability test was then performed at both time points (24 and 48 h).

### 2.3. Cell Viability Assay

Cell viability was assessed using a PrestoBlue™ Cell Viability Reagent (cat. nr A13261; Thermo Fisher Scientific). HGrC1, COV434 and KGN cells were cultured for 24 h and then exposed to T_3_, GW0742 or celecoxib. Medium containing DMSO (0.01%) was used as a control. In addition, the effect of pre-treatment with GW7042 and celecoxib (10 µM) on the action of T_3_ (10 nM) in HGrC1, COV434 and KGN cells was assessed. After 24 or 48 h, fluorescence was measured in a spectrofluorometer (FLx800, BioTek Instrument, Winooski, VT, USA) at an excitation wavelength of 530 nm and an emission wavelength of 590 nm. The results were analysed by KJ Junior software; KC4 (BioTek Instruments).

### 2.4. Real-Time PCR

Real-time PCR was conducted to assess the expression of the following genes: *THRA* (Hs00268470_m1), *THRB* (Hs00230861_m1), *PPARD* (Hs04187066_g1) and *PTGS2* (Hs00153133_m1), as described in our earlier study [19]. The RT-qPCR reaction was performed using control cells and cells treated with T_3_ (1 nM), GW0742 (10 µM) or celecoxib (10 µM) for 24 h to reflect the temporal sequence of events within the cell following exposure to the T_3_ hormone. The reaction was executed using the TaqMan TM Gene Expression Cells-to-CTTM kit (4399002; Applied Biosystems/Thermo Fisher Scientific, Waltham, MA, USA). Expression was normalised to that of GAPDH (4310884E), and relative expression was assessed using the 2^−ΔΔCt^ method [20].

### 2.5. Western Blot Analysis

First, cells were harvested, washed with phosphate-buffered saline (PBS) and lysed on ice. Next, the lysate was centrifuged at 4 °C/15,000 rpm for 15 min and the supernatant collected. The total protein concentration was quantified using a BCA protein assay kit (23225, Thermo Fisher Scientific). Proteins were separated in Mini-PROTEAN TGX Precast Protein Gels (Bio-Rad, Hercules, CA, USA) and then transferred to polyvinylidene difluoride membrane packs (Bio-Rad) using the Trans-Blot Turbo transfer system (Bio-Rad). The membranes were then incubated with antibodies specific for TRα (STJA0003800) and TRβ (STJA0003803) (St John’s Laboratory Ltd., London, UK). β-actin (A5316, Sigma-Aldrich) was used as a loading control. Bands were visualised using Western Bright Sirius Western blotting HRP substrate (Advansta, Menlo Park, CA, USA). Protein bands from three independent experiments were evaluated by densitometry using VisionWorks LS Acquisition, and Analysis software (https://www.labortechnik.com/en/analysis-software-visionworksls, accessed on 1 December 2024, UVP, Upland, CA, USA).

### 2.6. Seahorse Analysis

The oxygen consumption rate (OCR), the extracellular acidification rate (ECAR) and the ATP production rates from mitochondrial respiration (mitoATP) and glycolysis (glycoATP) were measured using a Seahorse XFp Analyzer (Agilent, Santa Clara, CA, USA) with the Seahorse XFp Real-Time ATP Rate Assay Kit (Agilent, cat#: 103591-100) following the manufacturer’s instructions. Briefly, HGrC1 and KGN cells were seeded in 8-well Seahorse assay microplates and cultured overnight for attachment. The cells were treated for 24 h with T_3_ (10 nM), GW0742 (10 µM) or T_3_ (10 nM) together with GW0742 (10 µM) for 24 h. Prior to the assay, cells were washed, and the medium was replaced with Seahorse XF DMEM supplemented with 10 mM glucose, 1 mM sodium pyruvate and 2 mM L-glutamine. ECAR and OCR measurements were made after the addition of T_3_ (10 nM) or GW0742 (10 µM), followed by oligomycin (1.5 µM) and, finally, rotenone and antimycin A (Rot/AA (0.5 µM). XFp assays were performed in triplicate. Metabolic activity was analysed both in real time at the time of the reagent addition and after 24 h exposure to the test compound. The aim of the study was to determine whether changes in the metabolic activity reprogram cells to alter their viability. The protein level of each well was quantified to normalise readings from the Seahorse XF Analyzer. The cells were lysed using RIPA Lysis Buffer (#89900, ThermoFisher Scientific) mixed with protease inhibitor (#1862209, ThermoFisher Scientific) according to the manufacturer’s protocol; the protein level was measured using a Nanodrop spectrophotometer (DeNovix, DS-11, Wilmington, DE, USA). The data were analysed using Seahorse Wave software; https://www.agilent.com/en/product/cell-analysis/real-time-cell-metabolic-analysis/xf-software/seahorse-xfp-analyzer-software-740905 (accessed on 1 December 2024) (Seahorse Bioscence, North Billerica, MA, USA).

### 2.7. Synergy Quotient Calculation for Synergism

The synergism quotient (SQ) was calculated by subtracting the baseline values from all treatments: GW0742, celecoxib or T_3_ and then dividing the effects of combined treatments by the sum of individual treatments. A SQ greater than 1.0 indicates a synergism for a given measured response.

### 2.8. Statistical Analysis

Data represent the mean ± SD of three independent experiments, all performed in independent triplicates. Statistical analysis was carried out by one-way or two-way ANOVA, followed by Tukey’s test (GraphPad Software Inc. 8.0.1, San Diego, CA, USA). The level of significance was set at * *p* < 0.05, ** *p* < 0.01 or *** *p* < 0.001.

## 3. Results

### 3.1. Basal Expression of TRα and TRβ in Healthy Ovarian Granulosa Cells (HGrC1) and a Rare Type of Ovarian Cancer Cells (COV434 and KGN)

**Figure 1:**
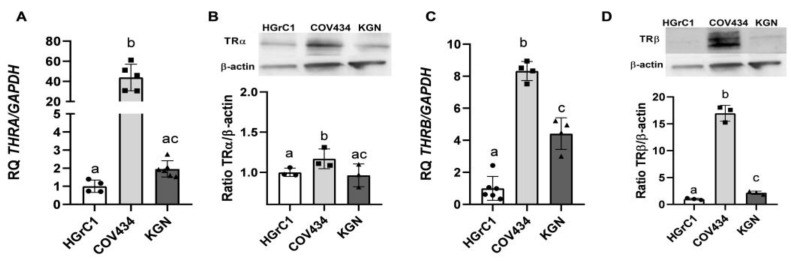
Basal expression profile of TRα and TRβ. Basal mRNA and protein expression of TRα (*THRA*) (**A**,**B**) and TRβ (*THRB)* (**C**,**D**) in HGrC1, COV434 and KGN cells. The expression level of *THRA* and *THRB* in HGrC1 cells was set to 1.0 RQ. Each bar represents the mean ± SD of three independent experiments. Statistically significant differences are denoted by mean values not sharing letters (*p* ≤ 0.05). The uncropped bolts are shown in Supplementary Materials.

First, we asked whether non-luteinised ovarian granulosa cells and a rare type of ovarian cancer cells express TRα and TRβ. Basal expression of *THRA* (TRα) and *THRB* (TRβ) in HGrC1, COV434 and KGN cells was measured at the mRNA level by qRT-PCR and at the protein level by Western blot analysis. The highest mRNA expression of TRα and TRβ was observed in COV434 cells (44-fold and 8.3-fold, respectively, vs. HGrC1 cells) (Figure 1A,C). Furthermore, the expression of TRβ was significantly higher in KGN cells (4.4-fold) than in HGrC1 cells (Figure 1C). The same TRα and TRβ expression profile was observed at the protein level (Figure 1B–D); however, differences in the expression of TRα protein were not as large as those observed for mRNA (Figure 1B).

### 3.2. Effect of T_3_ on the Viability and Metabolic Activity of Non-Luteinised Ovarian Granulosa Cells

**Figure 2:**
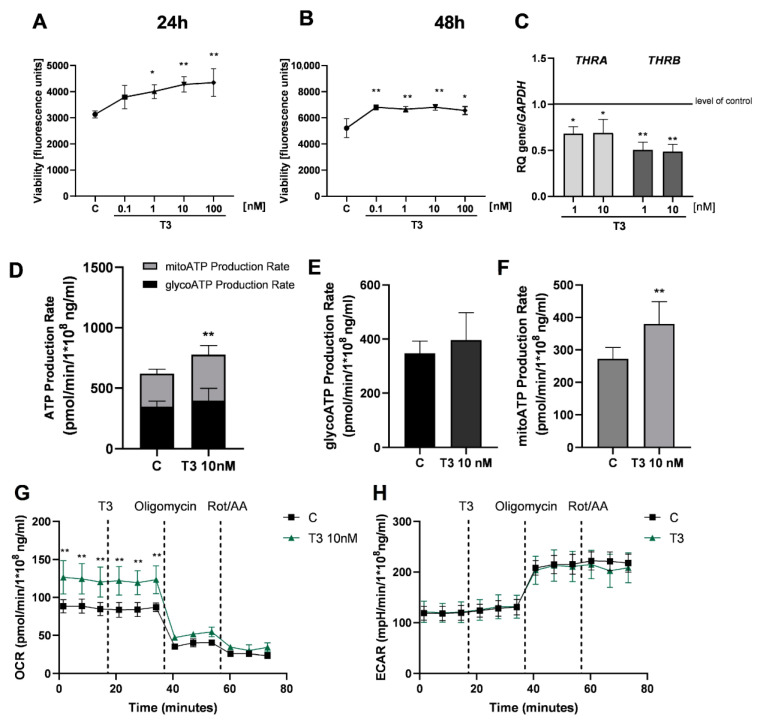
Effect of triiodothyronine (T3) on the viability and metabolic activity of non-luteinised ovarian granulosa cells. Dose-dependent effects of T3 (0.1, 1, 10 and 100 nM) on the viability of HGrC1 cells after 24 h (**A**) and 48 h (**B**) of treatment. C, control (0.01% DMSO, dimethyl sulfoxide). RFU, relative fluorescence units. Effect of T3 (1 and 10 nM) on the expression of TRα and TRβ mRNA in HGrC1 cells (**C**) after 24 h. mRNA expression by vehicle-treated cells was set to 1.0. RQ, relative quantity. Total ATP production rates in HGrC1 cells after stimulation of T3 (10 nM) (**D**). ATP production rates from glycolysis respiration (glycoATP) (**E**) and mitochondrial (mitoATP) (**F**) in HGrC1 cells after treatment with T3 (10 nM). Oxygen consumption rates (OCRs) (**G**) and extra cellular acidification rates (ECARs) (**H**) during the challenge with T3 (10 nM) in HGrC1 cells. Data represent the mean ± SD of three independent experiments. * *p* < 0.05, ** *p* < 0.01.

Since analyses showed that HGrC1 cells express *THRA* and *THRB*, we analysed the viability and metabolism of these cells under the influence of T_3_. T_3_ increased the viability of HGrC1 cells in a dose-dependent (0.1–100 nM) manner compared to untreated control cells. The viability of HGrC1 cells increased after treatment for 24 h with T_3_ at 1, 10 and 100 nM (1.28-, 1.37- and 1.38-fold, respectively, vs. the control group; Figure 2A; *p* < 0.05 and *p* < 0.01) and 48 h (1.3-, 1.27-, 1.32- and 1.28- fold, respectively, vs. the control group; Figure 2B; *p* < 0.05 and *p* < 0.01). Furthermore, we analyzed the effect of T_3_ on the expression of *THRA* and *THRB* in HGrC1 cells. T_3_ (1 and 10 nM) decreased the mRNA expression of TRα and TRβ (1.5- and 1.48-fold and 1.99- and 2.1-fold, respectively) in a dose-dependent manner (Figure 2C, *p* < 0.05 and *p* < 0.01). The total ATP level and mitoATP levels were significantly higher in HGrC1 after treatment with T_3_ (10 nM) after 24 h (Figure 2D,F; *p* < 0.01), but we did not observe differences in the glycoATP production rate (Figure 2E). Compared to the control group, cells after treatment with T_3_ (10 nM) exhibited a change in OCR, which revealed that the mitochondrial pathway was significantly enhanced (Figure 2G; *p* < 0.01). ECAR results showed that the glycolytic pathway was not enhanced after treatment with T_3_ (10 nM) (Figure 2H).

### 3.3. Effect of T_3_ on the Viability and Metabolic Activity of Rare Ovarian Cancer Cells

**Figure 3:**
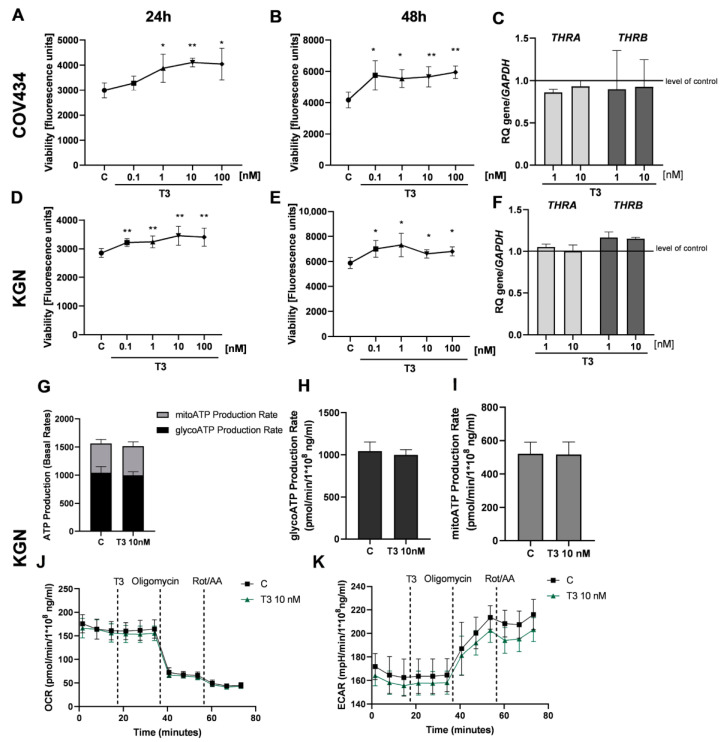
The effect of Triiodothyronine (T3) on viability and metabolic activity rare types of ovarian cancer cells. Dose-dependent effects of T3 (0.1, 1, 10 and 100 nM) on the viability of COV434 and KGN cells after 24 h (**A**,**D**) and 48 h (**B**,**E**) of treatment. C, control (0.01% DMSO; dimethyl sulfoxide). RFU, relative fluorescence units. Effect of T3 (1 and 10 nM) on the expression of TRα and TRβ mRNA in COV434 cells (**C**) and KGN cells (**F**) after 24 h. mRNA expression by vehicle-treated cells was set to 1.0. RQ, relative quantity. Total ATP production rates in KGN after stimulation with T3 (10 nM) (**G**). ATP production rates from glycolysis respiration (glycoATP) (**H**) and mitochondrial (mitoATP) (**I**) in KGN cells after treatment with T3 (10 nM). Oxygen consumption rates (OCRs) (**J**) and extra cellular acidification rates (ECAR) (**K**) during challenge with T3 (10 nM) in KGN cells. Data represent the mean ± SD of three independent experiments. * *p* < 0.05, ** *p* < 0.01.

Because HGrC1 cells expressing *THRA* and *THRB* responded to T_3_ hormones in the next step, we analyzed the effect of T_3_ on rare ovarian cancer cells, which also express *THRA* and *THRB.* Similarly, T_3_ increased the viability of COV434 after 24 h (1.29-, 1.37-, and 1.35-fold, respectively, vs. the control group; Figure 3A, *p* < 0.05 and *p* < 0.01) and 48 h (1.38-, 1.33-, 1.36- and 1.43-, respectively, vs. the control group) (Figure 3B, *p* < 0.05 and *p* < 0.01), as well as KGN cells after 24 h (1.13-, 1.14-, 1.21- and 1.19-fold, respectively, vs. the control group) and 48 h (1.19-, 1.24-, 1.12- and 1.16-fold vs. the control group; Figure 3D, *p* < 0.01; Figure 3E, *p* < 0.05). We also analyzed the effect of T_3_ on the expression of *THRA* and *THRB* in COV434 and KGN cells. T_3_ (1 and 10 nM) had no effect on mRNA expression of TRα and TRβ in these cells (Figure 3C,F). Furthermore, we did not observe a change in the metabolic activity of KGN cells after treatment with T_3_ (10 nM). The total ATP, mitoATP and glycoATP production rates were not significantly different in KGN cells after treatment with T_3_ (10 nM) after 24 h (Figure 3G–I). The OCR and ECAR results were not enhanced after treatment of KGN cells with T_3_ (10 nM) (Figure 3J,K).

### 3.4. Effect of TRα and TRβ Antagonist (GW0742 and Celecoxib) on the Viability and Metabolic Activity of Non-Luteinised Ovarian Granulosa Cells

**Figure 4:**
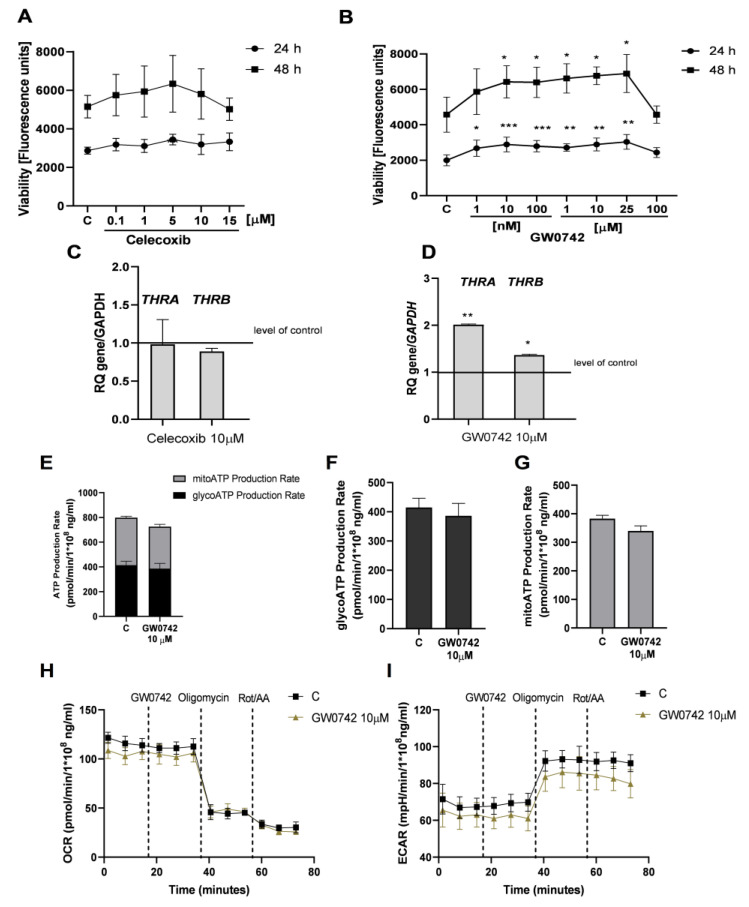
The effect of GW0724 and celecoxib on the viability and/or metabolic activity of non-luteinised ovarian granulosa cells. Dose-dependent effects of celecoxib (0.1–15 µM) (**A**) and GW0742 (0.001–25 µM) (**B**) on the viability of HGrC1 cells after 24 and 48 h. C, control (0.01% DMSO; dimethyl sulfoxide). RFU, relative fluorescence units. Effects of celecoxib (10 µM) (**C**) and GW0742 (10 µM) (**D**) on mRNA expression of TRα, TRβ in HGrC1 cells after 24 h. mRNA expression in vehicle-treated cells was set to 1.0. RQ, relative quantity. ATP production rates of HGrC1 after stimulation with GW0742 (10 µM) (**E**). ATP production rates from glycolysis respiration (glycoATP) (**F**) and mitochondrial respiration (mitoATP) (**G**) in HGrC1 cells after treatment with GW0742 (10 µM). Oxygen consumption rates (OCRs) (**H**) and extra cellular acidification rates (ECARs) (**I**) during the challenge with GW0742 (10 µM) in HGrC1 cells. Data represent the mean ± SD of three independent experiments. * *p* < 0.05, ** *p* < 0.01 and *** *p* < 0.001.

Zloh et al. showed that celecoxib antagonises TRβ; however, GW0742 can bind and antagonise TRα and TRβ [15,16]. Firstly, we analysed the effect of celecoxib on the viability of non-luteinised ovarian granulosa cells expressing TRβ. The viability of HGrC1 cells after treatment with celecoxib for 24 h and 48 h remained unchanged (Figure 4A). mRNA expression of TRα and TRβ in HGrC1 cells after treatment with 10 µM celecoxib was not significantly different (Figure 4C). On the other hand, we assessed that GW0742 (0.001–25 µM) increased the viability of HGrC1 cells after 24 h (1.34-, 1.45-, 1.40-, 1.36-, 1.45- and 1.52-fold, respectively, vs. the control; Figure 4B; *p* < 0.05, *p* < 0.01 and *p* < 0.001) and after 48 h (1.40-, 1.40-, 1.45-, 1.48- and 1.51-fold vs. the control; Figure 4B; *p* < 0.05). Additionally, we examined the mRNA expression of TRα and TRβ after treatment with GW0742 at a dose of 10 µM. GW0742 increased mRNA expression of TRα and TRβ in HGrC1 cells (1.73- and 1.36-fold, respectively; Figure 4D; *p* < 0.05 and *p* < 0.01). Moreover, GW0742 (10 µM) did not alter the metabolic activity of HGrC1 cells. The total ATP, mitoATP and glycoATP production rates were not significantly different in HGrC1 cells after treatment with GW0742 (10 µM) after 24 h (Figure 4E–G). The OCR and ECAR results were not enhanced after treatment of HGrC1 cells with GW0742 (10 µM) (Figure 4H,I).

### 3.5. Effect of TRα and TRβ Antagonist (GW0742 and Celecoxib) on the Viability and Metabolic Activity in a Rare Type of Ovarian Cancer Cells

**Figure 5:**
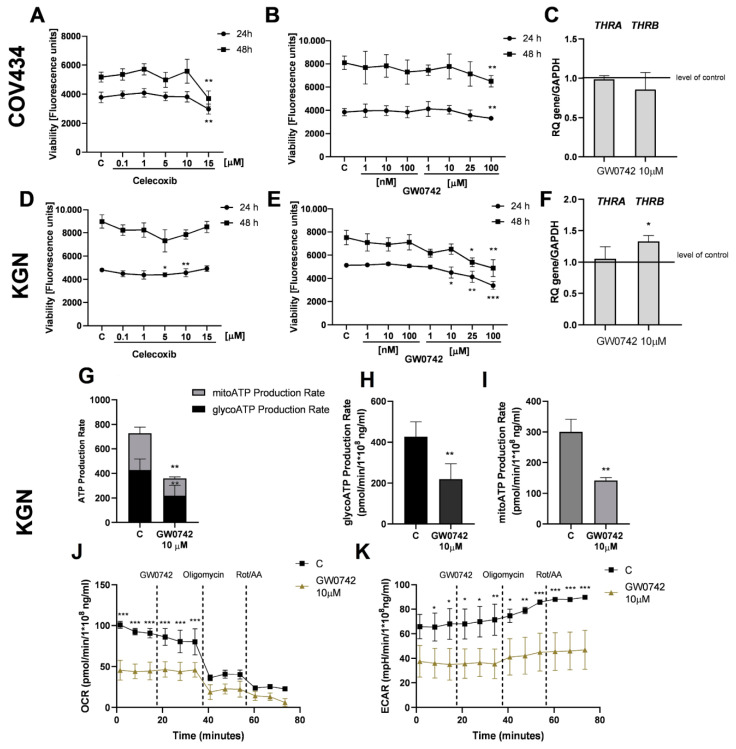
The effect of GW0724 and celecoxib on the viability and/or metabolic activity of a granulosa cell tumour (GCT). Dose-dependent effects of celecoxib (0.1–15 µM) (**A**,**D**) and GW0742 (0.001–25 µM) (**B**,**E**) after 24 and 48 h treatment on the viability of COV434 and KGN cells, respectively. C, control (0.01% DMSO; dimethyl sulfoxide). RFU, relative fluorescence units. Effects of GW0742 (10 µM) (**C**,**F**) on the mRNA expression of TRα and TRβ in COV434 and KGN cells after 24 h, respectively. mRNA expression in vehicle-treated cells was set to 1.0. RQ, relative quantity. ATP production rates of KGN cells after stimulation with GW0742 (10 µM) (**G**). ATP production rates from glycolytic respiration (glycoATP) (**H**) and mitochondrial (mitoATP) (**I**) in KGN cells after treatment with GW0742 (10 µM). Oxygen consumption rates (OCRs) (**J**) and extra cellular acidification rates (ECARs) (**K**) during the challenge with GW0742 (10 µM) in KGN cells. Data represent the mean ± SD of three independent experiments. * *p* < 0.05, ** *p* < 0.01 and *** *p* < 0.001.

The stimulation cells of celecoxib (15 µM) reduced the viability of COV434 cells after 24 and 48 h (1.27- and 1.40-fold, respectively; Figure 5A; *p* < 0.01 and *p* < 0.001). However, GW0742 (100 µM) decreased the COV434 cell viability after 24 and 48 h (1.16- and 1.25-fold, respectively; Figure 5B; *p* < 0.01). Additionally, GW0742 (10 µM) did not change the expression of *THRB* in COV434 cells (Figure 5C; *p* < 0.01). While the viability of KGN cells was decreased after treatment with celecoxib only in doses of 5 and 10 µM for 48 h (1.2- and 1.14-fold, respectively; Figure 5D; *p* < 0.05 and *p* < 0.01), the viability of KGN cells was decreased after treatment with GW0742 at doses of 10, 25 or 100 µM after 24 h (1.14-, 1.24- and 1.51-fold, respectively) and at dose 25 and 100 µM after 48 h (1.40- and 1.54-fold, respectively; Figure 5E; *p* < 0.05, *p* < 0.01 and *p* < 0.01). Additionally, GW0742 (10 µM) increased the expression of *THRB* in KGN cells (1.35-fold; Figure 5F; *p* < 0.05). Furthermore, GW0742 decreased the metabolic activity of KGN cells. The total ATP level and the glycoATP and mitoATP production rates were significantly lower in KGN cells after treatment with GW0742 (10 µM) after 24 h (Figure 5G–I; *p* < 0.01). Compared to the control group, cells after treatment with GW0742 (10 µM) exhibited a change in the OCR and ECAR, which revealed that the mitochondrial and glycolytic pathways were significantly reduced in KGN cells (Figure 5J,K; *p* < 0.05, *p* < 0.01 and *p* < 0.001). Celecoxib is well known as a COX-2 inhibitor; therefore, we analysed the basal expression of *PTGS2*. Interestingly, the HGrC1 cells did not express *PTGS2*, whereas the COV434 and KGN cells did. Furthermore, the expression of *PTGS2* was significantly higher in COV434 cells than in KGN cells (Figure S1A; *p* < 0.001). Moreover, celecoxib (10 µM) increased the expression of *PTGS2* in COV434 cells (1.49-fold) and in KGN cells (1.46-fold) (Figure S1B,C; *p* < 0.01). Because the main action of GW0742 is to antagonise the PPARβ/δ receptor, we assessed the basal expression of the PPARβ/δ receptor (*PPARD*) mRNA in HGrC1, COV434 and KGN cells. All three cell lines expressed PARβ/δ mRNA, with COV434 cells showing the highest expression (Figure S2A; *p* < 0.01). GW0742 increased the mRNA expression of PPARβ/δ in HGrC1 cells (2.26-fold, Figure S2B; *p* < 0.001) but did not impact the mRNA expression of PPARβ/δ in COV434 and KGN cells (Figure S2C,D).

### 3.6. GW0742 and Celecoxib Abolish the Stimulatory Effects of T_3_ on a Rare Type of Ovarian Cancer Cells

**Figure 6:**
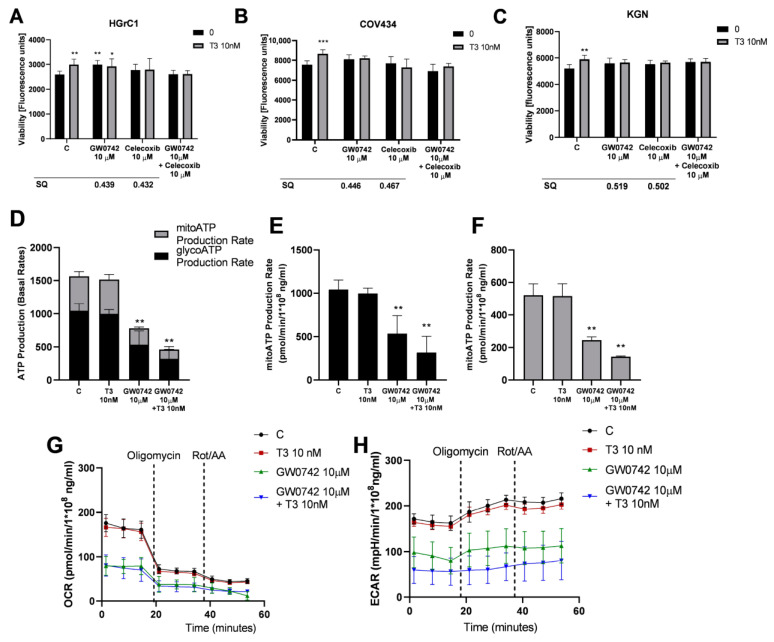
The effect of GW074 and celecoxib on the viability and metabolic activity in HGrC1, COV434 and KGN stimulated by T_3_. The effects of the addition of triiodothyronine (T_3_) (10 nM) in combination with pretreatment with the TRα and TRβ antagonists (GW0742) (10 µM) or a TRβ antagonist (celecoxib) (10 µM) for 48 h on the viability of HGrC1 (**A**), COV434 (**B**) and KGN cells (**C**). C, control (0.01% DMSO; dimethyl sulfoxide); RFU, relative fluorescence units. Synergism quotient (SQ) values were calculated to determine if there was any synergistic activity in the treatment with GW0742 or celecoxib with T_3_. ATP production rates of KGN cells after stimulation with T_3_ (10 nM), GW0742 (10 µM) and with T_3_ (10 nM) in combination with the pretreatment with GW0742 (10 µM) (**D**). ATP production rates from glycolysis respiration (glycoATP) (**E**) and mitochondrial respiration (mitoATP) (**F**) in KGN cells after treatment with T_3_ (10 nM), GW0742 (10 µM) and with T_3_ (10 nM) in combination with the pretreatment with GW0742 (10 µM). Oxygen consumption rates (OCRs) (**G**) and extra cellular acidification rates (ECARs) (**H**) during the challenge with T_3_ (10 nM), GW0742 (10 µM) and with T_3_ (10 nM) in combination with the pretreatment with GW0742 (10 µM) in KGN cells. Data represent the mean ± SD of three independent experiments. * *p* < 0.05, ** *p* < 0.01 and *** *p* < 0.001.

Because T_3_ increased the viability of HGrC1, COV434 and KGN cells in a dose-dependent manner, we next asked whether GW0742 (a TRα and TRβ antagonist) and celecoxib (a TRβ antagonist) abolish the effects of T_3_ in these cells. Pretreatment of cells with GW0742 (10 µM) and celecoxib (10 µM), either individually or together, abolished the stimulatory effect of T_3_ on the viability of COV434 and KGN cells (Figure 6B,C; *p* < 0.001). However, the stimulatory effect of T_3_ (10 nM) on the viability of HGrC1 was abolished after pretreatment with celecoxib (10 µM) (Figure 6A; *p* < 0.05; *p* < 0.01). Additionally, we observed an antagonistic effect at 10 μM of GW0742 with T_3_ (10 nM), with a SQ of 0.439, 0.446 and 0.519 in HGrC1, COV434 and KGN cells, respectively. Similarly, the antagonistic effect of celecoxib (10 μM) with T_3_ (10 nM) was detected in HGrC1, COV434 and KGN cells with a SQ of 0.432, 0.467 and 0.502, respectively (Figure 6A–C), Furthermore, pretreatment with GW0742 before T_3_ stimulation of KGN cells inhibited the metabolic activity of KGN cells. The total ATP level and the glycoATP and mitoATP production rates were significantly lower in KGN cells after stimulation with T_3_ (10 nM) in combination with the pretreatment with GW0742 (10 µM) after 24 h (Figure 6D–F; *p* < 0.01). Compared to the control group, cells after treatment with T_3_ (10 nM) in combination with the pretreatment with GW0742 (10 µM) exhibited a change in the OCR and ECAR, which revealed that the activities of the mitochondrial and glycolytic pathways were significantly reduced in KGN cells (Figure 6G,H; *p* < 0.001).

## 4. Discussion

The TRα and TRβ genes were first identified in human granulosa cells and ovarian stromal cells by Wakim et al. [2,3]. In addition, Aghajanova et al. showed that human granulosa cells in the antral follicles express TRα1 and TRβ1 proteins [21]. Furthermore, epidemiological data underscore the association between thyroid dysfunction and an increased incidence of ovarian cancer, as well as increased mortality in patients diagnosed with ovarian cancer [22]. Furthermore, the expression and localisation of TRα and TRβ determine the overall survival in epithelial OC patients [5,6]. Here, using cellular models, we showed that both rare OC, COV434 and KGN, express TRα and TRβ. Here, we show that rare types of OC express higher levels of *THRA* and *THRB* than non-luteinised ovarian granulosa cells. Moreover, we indicated that the mRNA expression of TRα and TRβ is the highest in COV434 cells compared to KGN cells. A study by Alexiadis et al. supported our observations, using nuclear receptor profiling of GCT to show that GCT express TRs [23].

A previous study reported that T_3_ levels in healthy women ranged from 0.8 to 1.6 ng/mL, whereas those in patients with hyperthyroidism were 5.7 ± 3.5 ng/mL and those in patients with hypothyroidism ranged from undetectable to 0.8 ng/mL [24]. Furthermore, T_3_ has been detected in ovarian follicular fluid at levels of 1.10 ± 0.14 ng/mL [3]. The expression of nuclear receptors for TRs determines the activity of TRs in target tissues. The presence of T_3_ in follicular fluid and TRs in rare types of OC suggests that T_3_ regulates processes in these cells. Silva et al. suggested that T_3_ has a direct effect on ovarian tissue [25]. Here, we examined the viability of non-luteinised ovarian cells and a rare type of OC after exposure to T_3_ at physiological (1 nM) and non-physiological (0.1, 10 and 100 nM) doses. We observed a dose-dependent increase in the viability of HGrC1 cells after treatment with T_3_, a finding consistent with that of Zhang et al., who showed that T_3_ (1 nM) increased the activity of FSH by inhibiting apoptosis, thereby promoting the proliferation of granulosa cells harvested from rats [26]. Simultaneously, for the first time, we observed downregulated mRNA expression of TRα and TRβ and increased ATP production rates from mitochondrial respiration (mitoATP) in HGrC1 cells. Ing reported that hormones regulate the expression of their own receptor(s) by creating autoregulatory feedback loops [27]. Here, we found that physiological and non-physiological doses of T_3_ increased the viability of COV434 and KGN cells but did not regulate the expression of TRα and TRβ genes or the metabolic ATP production rate in these cells. Similarly, the growth of COV434 cells in the presence of T_3_ (1, 10 and 100 nM) was observed by Falzacappa et al. [28]. Furthermore, T_3_ and T_4_ regulate oestrogen pathways, increase angiogenesis and regulate the expression of genes that increase tumour proliferation [29].

Because rare types of OC (GCT and SCCOHT) express TRs, they may be a target for substances that antagonise TRs. Studies show that the synthetic compound GW0742 and the nonsteroidal anti-inflammatory drug celecoxib may have such properties. GW0742 is a PPARβ/δ receptor agonist in proof-of-concept clinical trials for the treatment of hypercholesterolemia and dyslipidaemia [30,31]. Perez Diaz et al. reported the antagonistic properties of GW0742 against TRα and TRβ and also that the binding affinity of GW0742 for TRα and TRβ may be higher than that of the agonist T_3_ [15]. Therefore, we analysed the effects of GW0742 on the viability and metabolic activity of non-luteinised ovarian granulosa cells and a rare type of OC. Treatment of non-luteinised ovarian granulosa cells with GW072 increased cell viability in dose-dependent manner without causing a change in the ATP production rate and simultaneously upregulated the expression of *THRA*, *THRB* and *PPRAD* in these cells. Interestingly, rare OC showed the opposite response to GW0742. We observed a decrease in the viability of COV434 cells after treatment with 100 µM GW0742 and of KGN cells after treatment with 25–100 µM. A stronger effect of GW0742 on the reduction of cell viability was observed in GCT. Moreover, GW0742 reduced the ATP production rate from mitochondrial respiration (mitoATP) and glycolysis (glycoATP) in KGN cells. No studies have examined the effect of GW0742 on OC cells, but GW0742 (1–10 μM) inhibits the growth of colon cancer HCT116 cells [32]. Similarly, Ma et al. showed that GW0742 (10 µM) increased apoptosis and inhibited the growth of human endometrial cancers (the Ishikawa, RL-95 and Sawano cell lines) [33]. Furthermore, we found that GW0742 upregulated mRNA expression of TRβ only in ovarian granulosa cells, without affecting mRNA expression of TRα and PPARβ/δ. Our findings suggest that GW0742 affects GCT by regulating the expression of TRβ. Indeed, loss of *THRB* gene expression has been described in many malignancies, including lung, melanoma, breast, head and neck, kidney, cervix, ovarian and testicular cancers [34]. Furthermore, TRβ is considered to be a potential tumour suppressor in the breast and thyroid [35,36]. We also found that COV434 cells expressed higher levels of PPARβ/δ than non-luteinised cells, a finding consistent with another study showing upregulation and localisation of PPAR β/δ in endometrial cancer cells [37].

Celecoxib is a selective COX-2 inhibitor that is used as an anti-inflammatory drug for the treatment of rheumatoid arthritis or osteoarthritis [38]. Interestingly, computational chemistry predictions indicated that celecoxib binds to TRβ [14]. Here, we found that celecoxib had no effect on the viability or mRNA expression of TRα and TRβ in non-luteinised ovarian granulosa cells. Furthermore, HGrC1 cells did not express COX-2. In contrast, celecoxib decreased the viability both of rare OC cell lines and increased the mRNA expression of COX-2. Earlier studies by Suri et al. indicated that celecoxib inhibited the proliferation of serous OC cells in vitro, as well as tumours in a mouse model [39]. Studies suggest that celecoxib works by blocking the function and disrupting the production of COX-2 by serous OC and breast cancer cells [39,40]. When we compared the expression of COX-2 in the three cell lines, we did not detect mRNA expression of COX-2 in HGrC1 cells. This is consistent with studies showing that normal ovarian epithelium does not express COX-2, whereas a high expression of COX-2 can be detected in ovarian cancer cells [41,42,43]. Furthermore, we observed that COV434 expressed higher levels of PTGS2 than KGN cells. These findings agree with a previous report showing that overexpression of COX-2 is associated with a poor prognosis of breast, prostate and OC [44,45]. Menczer et al. showed that human GCTs were positive for COX-2 [46], whereas another study showed that COX-2 enhances tumour progression by increasing cell proliferation, inhibiting apoptosis and promoting angiogenesis and invasion [47]. However, we suggest that celecoxib decreased the viability of GCT less than GW0742.

Additionally, we found that pretreatment with celecoxib/GW0742, either alone or together, abolished the effects of T_3_ on the viability of a rare type of OC. Furthermore, the reduction in metabolic activity in ovarian GCT by a pretreatment with GW0742 disabled the action of T_3_. These data suggest that TR inhibitors can protect against the negative effects of T_3_ in rare types of ovarian cancer.

Interestingly, our analysis showed a relationship between the regulation of TRα and TRβ expression in normal and rare OC cells. Downregulation of TRα and TRβ expression in healthy ovarian granulosa cells is correlated with an increase of metabolic activity, mainly in the mitochondrial respiration pathway. In the absence of changes or upregulation of TRα and TRβ expression (higher TRα/TRβ ratio), disturbances in the metabolic activity of granulosa cells and rare OC were not observed. In contrast, an upregulated TRβ expression, a TRα/TRβ ratio in favour of TRβ, was accompanied by a reduction in the metabolic activity of rare OC. Thus, these data may indicate that, potentially, the metabolic activity of ovarian GCs and rare OCs is conditioned by the level of TR expression.

## 5. Conclusions

In conclusion, we show here that rare OC cell lines express higher levels of TRα and TRβ than non-luteinised ovarian GCs cell lines. However, our data showed that their ratio is more important than their expression level. We demonstrated that T_3_ increases the viability of non-luteinised ovarian GCs and rare OC at both physiological and non-physiological doses. Interestingly, we found that the expression of TRα and TRβ after T_3_ stimulation differs between rare OC and non-luteinised ovarian GCs, which is probably due to the lack of autoregulation of the feedback loops during TR signalling. Our data provide novel insight into the growth inhibitory activities of GW0742 in ovarian GCT expressing TRα and TRβ. More importantly, we show that GW0742 upregulate TRβ, which is thought to be a tumour suppressor. Additionally, GW0742 reduces the metabolic activity of GCT. These findings suggest that GW0742, which reduces viability and metabolic activity while upregulating TRβ expression, can be a new perspective for treating GCT expressing TRα and TRβ as an adjuvant therapy. However, further in vitro and clinical studies are needed.

## Data Availability

The data presented in this study are available on request from the corresponding author.

## Supplementary Materials

The following supporting information can be downloaded at https://www.mdpi.com/article/10.3390/cancers16234069/s1: Figure S1: Basal and celecoxib-induced COX-2 expression profile in HGrC1, COV434 and KGN. Figure S2: Basal and GW074-induced PPARβ/δ expression profile in HGrC1, COV434 and KGN. Western blot File.
