# Supplementary figures and images for: GW0742 as a Potential TRα and TRβ Antagonist Reduces the Viability and Metabolic Activity of an Adult Granulosa Tumour Cell Line and Simultaneously Upregulates TRβ Expression
Source: Cancers (Basel). 2024 Dec 5;16(23):4069. doi: 10.3390/cancers16234069 (PMC11640550; doi:10.3390/cancers16234069)

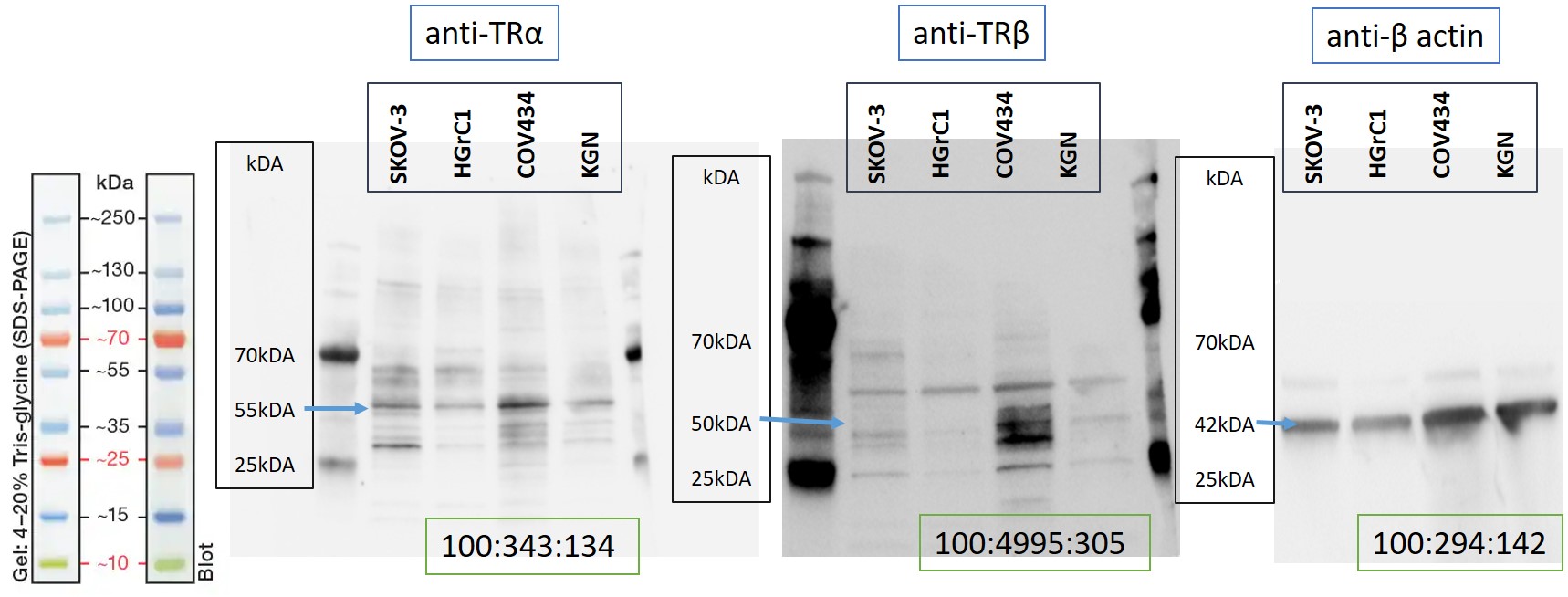

Supplement: Supplementary file 1 [file cancers-16-04069-s001.zip › cancers-3341363-western blot.jpg]

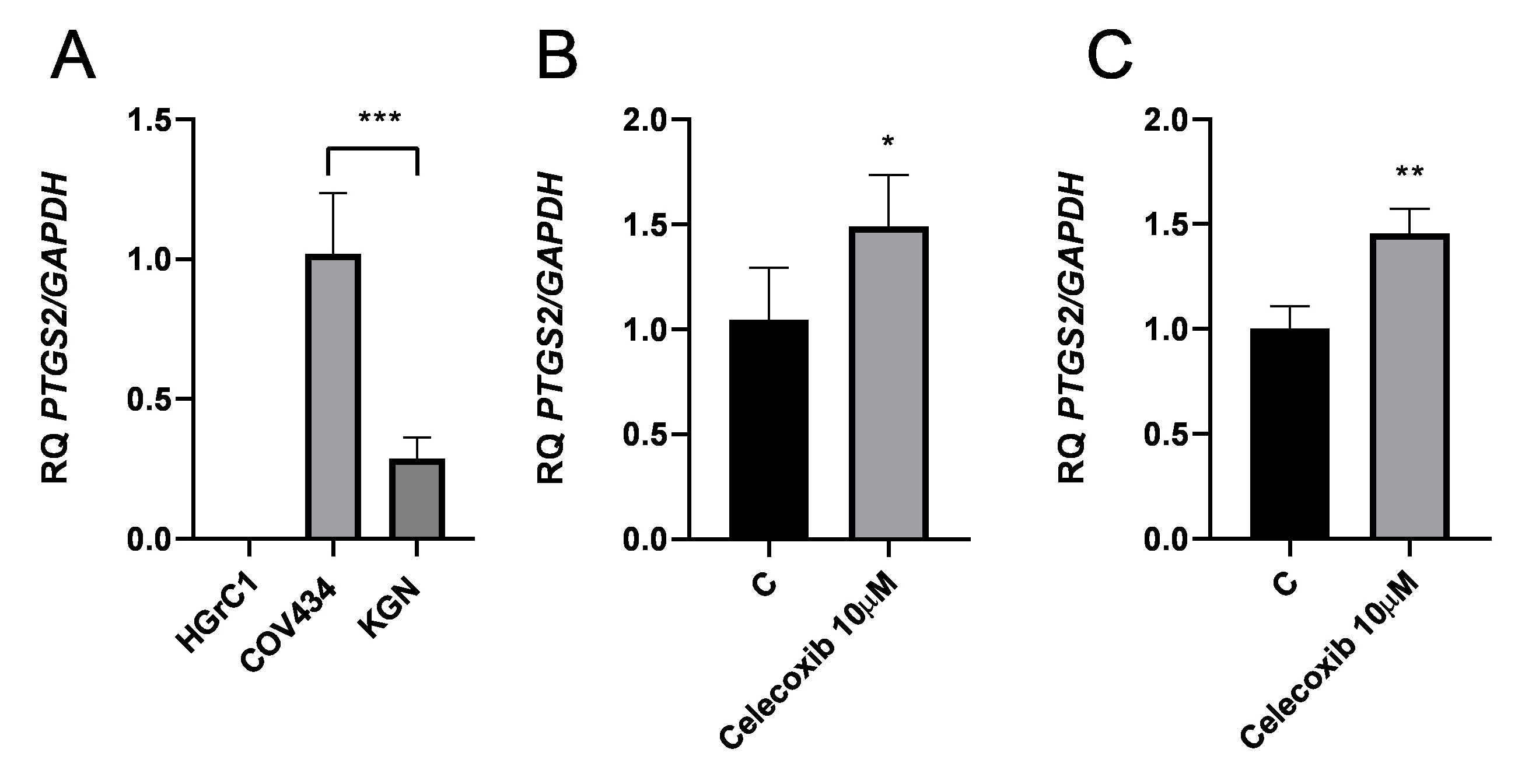

Supplement: Supplementary file 1 [file cancers-16-04069-s001.zip › Fig. S1.jpg]

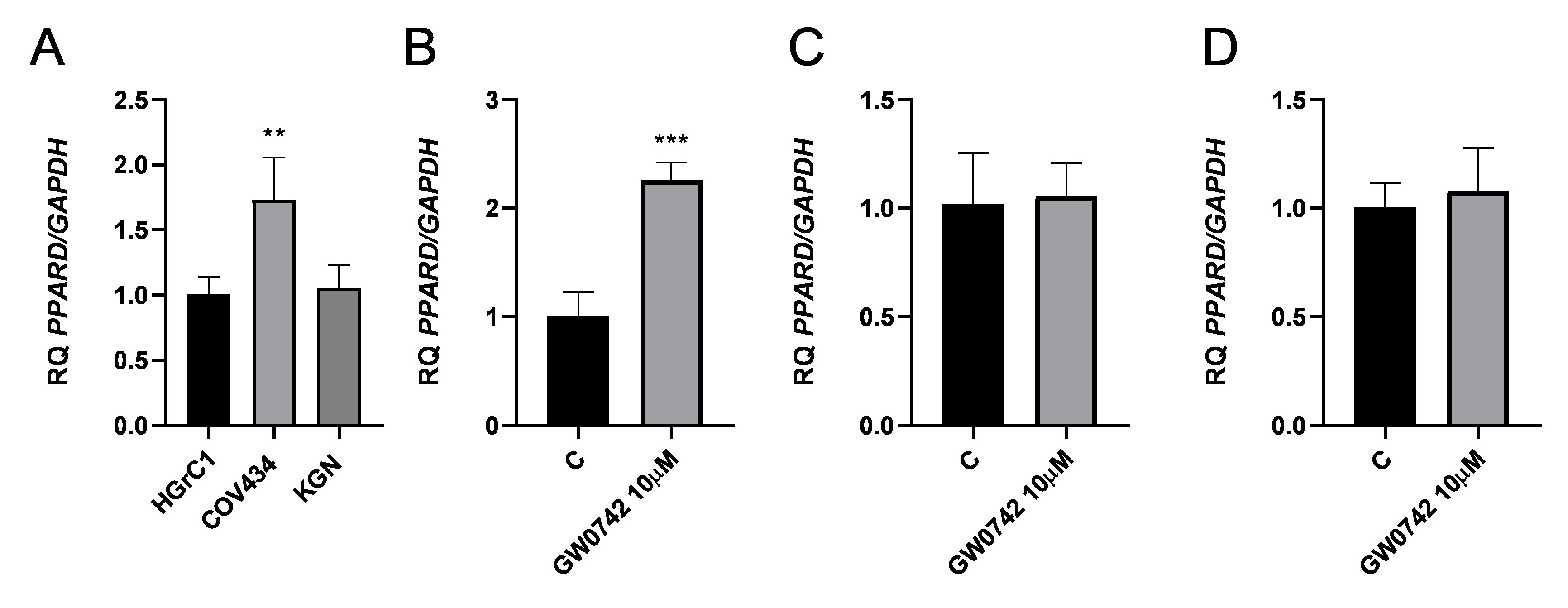

Supplement: Supplementary file 1 [file cancers-16-04069-s001.zip › Fig. S2.jpg]
